# Type 2 Diabetes Induces Mitochondrial Dysfunction in Zebrafish Skeletal Muscle Leading to Diabetic Myopathy via the miR-139-5p/NAMPT Pathway

**DOI:** 10.3390/ijms26020752

**Published:** 2025-01-17

**Authors:** Zhanglin Chen, Zuoqiong Zhou, Qinhua Deng, Yunyi Zou, Bihan Wang, Shuaiwang Huang, Jiaqi Tian, Lan Zheng, Xiyang Peng, Changfa Tang

**Affiliations:** State Key Laboratory of Developmental Biology of Freshwater Fish, Key Laboratory of Physical Fitness and Exercise Rehabilitation of Hunan Province, College of Physical Education, Hunan Normal University, Changsha 410012, China; zhanglinchen@hunnu.edu.cn (Z.C.); zhouzuoqiong@hunnu.edu.cn (Z.Z.); 202270153046@hunnu.edu.cn (Q.D.); 202020151346@hunnu.edu.cn (Y.Z.); wbhxhzz@hunnu.edu.cn (B.W.); 202320152962@hunnu.edu.cn (S.H.); jiaqitian@hunnu.edu.cn (J.T.); zheng@hunnu.edu.cn (L.Z.)

**Keywords:** exercise capacity, microRNA, mitochondrial dysfunction, myopathy, skeletal muscle, type 2 diabetes, zebrafish

## Abstract

Type 2 diabetes mellitus (T2DM) is a common metabolic disease that is frequently accompanied by multiple complications, including diabetic myopathy, a muscle disorder that is mainly manifested as decreased muscle function and reduced muscle mass. Diabetic myopathy is a relatively common complication among patients with diabetes that is mainly attributed to mitochondrial dysfunction. Therefore, we investigated the mechanisms underlying diabetic myopathy development, focusing on the role of microRNAs (miRs). Zebrafish were fed a high-sugar diet for 8 weeks and immersed in a glucose solution to establish a model of T2DM. Notably, the fish exhibited impaired blood glucose homeostasis, increased lipid accumulation in the skeletal muscles, and decreased insulin levels in the skeletal muscle. Additionally, we observed various symptoms of diabetic myopathy, including a decreased cross-sectional area of skeletal muscle fibers, increased skeletal muscle fibrosis, a significant decline in exercise capacity, and a significant decrease in mitochondrial respiratory function. Mechanistically, bioinformatic analysis combined with various molecular analyses showed that the miR-139-5p/NAMPT pathway was involved in long-term high-glucose-induced mitochondrial dysfunction in the skeletal muscle, leading to diabetic myopathy. Conclusively, this study provides a basis for the development of novel strategies for the prevention and treatment of diabetic myopathy.

## 1. Introduction

Diabetes mellitus (DM) is a chronic disorder that affects human health worldwide, with a significant proportion (10.5%) of adults aged 20–79 years affected by the disorder. Importantly, it is predicted that one in every eight adults (approximately 783 million people) will have DM by 2045 [1]. Type 2 diabetes mellitus (T2DM) is the most prevalent type of DM, accounting for more than 90% of all DM cases. Patients with T2DM exhibit poor responsiveness to insulin resulting in abnormal glucose metabolism. Insulin resistance primarily occurs in the skeletal muscle, liver, and adipose tissue, making these tissues unable to effectively utilize insulin and resulting in elevated blood glucose levels [2].

The skeletal muscle is the primary motor organ of the human body and is responsible for body movement and posture maintenance. Additionally, the skeletal muscle plays a crucial role in metabolism, particularly in glucose metabolism and energy balance. It regulates blood glucose levels through insulin-dependent and -independent mechanisms and promotes glucose uptake and utilization [3]. Although the skeletal muscle can influence glucose metabolism, disorders in glucose metabolism can also affect skeletal muscle function. Both type 1 and type 2 diabetes are characterized by a progressive decline in skeletal muscle mass and function, a condition known as diabetic myopathy [4,5]. In such cases, the skeletal muscle undergoes further structural, functional, and metabolic changes, including muscle fiber atrophy [6], alterations in myokine secretion [7], impaired mitochondrial structure and bioenergetics [8], fiber-type transformations, and reduced oxidase activity [9], resulting in decreased muscle strength [4], impaired functional capacity, and ultimately, increased mortality [10].

Mitochondrial function directly influences the metabolic capacity of the skeletal muscle. Notably, mitochondria are responsible for energy production and participate in the oxidation of fatty acids. Mitochondrial dysfunction can result in reduced fatty acid oxidation, resulting in fat accumulation in the skeletal muscle [11]. Additionally, mitochondria play a crucial role in regulating metabolic pathways and maintaining energy homeostasis in tissues. Diabetes is linked to decreased mitochondrial function, including reduced mitochondrial number [12], impaired lipid oxidation [13], and excessive production of reactive oxygen species (ROS) [14]. As in rodents, proteins related to the mitochondrial function in zebrafish have undergone extensive experimental manipulations. In particular, the mitochondrial dynamics between zebrafish and other animal models are extremely similar [15]. Diabetes may inhibit proper mitophagy, thus inducing the accumulation of damaged mitochondria in the skeletal muscle, resulting in energy metabolism disorders [16]. Additionally, diabetic and obese mice display mitochondrial dysfunctions, including reduced mitochondrial DNA copy number, an increased number of disordered cristae, and reduced citrate synthase activity [17]. Excess ROS generated by dysfunctional mitochondria can damage cellular components and activate stress-sensitive pathways, further impairing the insulin mechanism [18,19].

Nicotinamide phosphoribosyltransferase (NAMPT) is a crucial rate-limiting enzyme in the NAD salvage pathway and is dysregulated in metabolic diseases, such as diabetes [20,21]. NAMPT can regulate the pathogenesis of T2DM by influencing the oxidative stress response, apoptosis, lipid and glucose metabolism, inflammation, and insulin resistance [22]. NAMPT is widely expressed throughout the body, and mice lacking it exhibit embryonic lethality [23]. Mice with a muscle-specific deficiency of NAMPT exhibit myofiber degeneration and a loss of strength and endurance, whereas lifelong overexpression of NAMPT increases NAD^+^ levels and enhances physical function in aged mice [24]. Additionally, NAMPT knockdown in mouse C2C12 myoblasts reduced NAD^+^ levels and mitochondrial biogenesis [25]. Recent studies indicate that miRNAs are closely associated with various human diseases, including DM, a complex multifactorial metabolic disorder. In DM, exosomal miRNAs are taken up by recipient cells and exert biological functions, thereby regulating the progression of DM-related complications [26]. In human experiments, the plasma levels of miR-139-5p were elevated in individuals with high fasting blood glucose and insulin resistance [27]. However, the specific mechanism of action of miR-139-5p/NAMPT in diabetic myopathy remains unclear.

The model animal zebrafish exhibits similar structural and physiological characteristics to humans in regulating glucose homeostasis and energy metabolism [28,29]. Additionally, the skeletal muscle shares similar molecular (namely, the conserved molecular regulatory network governing myogenesis), histological, and ultrastructural characteristics with mammalian muscle. Therefore, we investigated the mechanisms underlying diabetic myopathy development, focusing on the role of microRNAs (miRs). Specifically, we constructed a zebrafish model of T2DM to explore the molecular mechanism by which type 2 diabetes induces mitochondrial dysfunction and diabetic myopathy in skeletal muscle via NAMPT. It is expected that this study will provide novel information for the prevention and treatment of diabetes and offer therapeutic targets in clinical settings.

## 2. Results

### 2.1. High-Glucose Treatment Induces T2DM in Zebrafish

**Figure 1:**
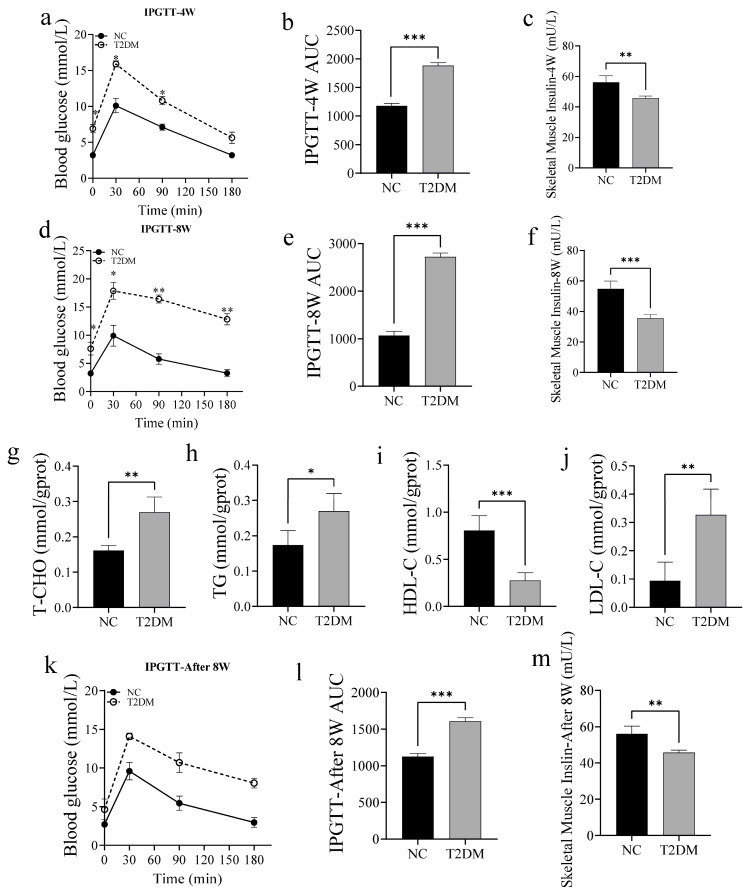
High-glucose treatment resulted in the generation of a zebrafish model of type 2 diabetes mellitus (T2DM). (**a**) Intraperitoneal glucose tolerance test (IPGTT) curve for zebrafish after 4 weeks of high-glucose treatment (0.5 mg/g body weight (BW)); (**b**) area under the glucose tolerance curve after 4 weeks of high-glucose treatment; (**c**) insulin content in skeletal muscle after 4 weeks of high-glucose treatment; (**d**) IPGTT curve for zebrafish after 8 weeks of high-glucose treatment; (**e**) area under the glucose tolerance curve after 8 weeks of high-glucose treatment; and (**f**) insulin content in skeletal muscle after 8 weeks of high-glucose treatment. (**g**) Total cholesterol (T-CHO); (**h**) triglyceride (TG); (**i**) high-density lipoprotein-cholesterol (HDL-C); and (**j**) low-density lipoprotein-cholesterol (LDL-C). (**k**) IPGTT curve for zebrafish after normal breeding for 8 weeks following high-glucose intervention for 8 weeks; (**l**) area under the glucose tolerance curve for zebrafish after normal breeding for 8 weeks following high-glucose intervention for 8 weeks; and (**m**) insulin content in skeletal muscle of zebrafish after normal breeding for 8 weeks following high-glucose intervention for 8 weeks. * *p* < 0.05, ** *p* < 0.01, *** *p* < 0.001. (*n* = 6 samples/group).

To explore the effect of high sugar levels on skeletal muscle function, zebrafish were fed a high-sugar diet and immersed in a glucose solution for 4 and 8 weeks. After the experiment, the fish were subjected to an intraperitoneal glucose tolerance test (IPGTT). Zebrafish were intraperitoneally injected with glucose (0.5 mg of glucose per gram body weight), and blood glucose concentrations were measured at 0, 30, 90, and 180 min post-injection. At 4 weeks, blood glucose concentrations peaked at 30 min post-glucose injection in the NC and T2DM groups, but gradually returned to pre-injection levels within 180 min. However, blood glucose levels were significantly higher in the T2DM group than in the NC group at all time points (Figure 1a,b). Although blood glucose levels in the T2DM group decreased over time after 8 weeks of intervention, the levels at 30, 90, and 180 min post-glucose injection were still significantly higher in the T2DM group than in the NC group (Figure 1c,d). Additionally, the rate of decline in blood glucose levels was significantly lower in the T2DM group than in the NC group. Moreover, insulin levels in the skeletal muscle decreased with increasing duration of high-glucose intervention in the T2DM group (Figure 1e,f). Therefore, the 8-week high-glucose intervention was selected for subsequent experiments.

Considering that hyperglycemia can cause lipid metabolism disorder, we examined the effect of T2DM on lipid metabolism in the skeletal muscle of zebrafish. Specifically, we measured the total cholesterol (T-CHO), triglycerides (TG), low-density lipoprotein-cholesterol (LDL-C), and high-density lipoprotein-cholesterol (HDL-C) levels in the skeletal muscle. Compared with those in the NC group, there was a significant increase in T-CHO, TG, and LDL-C levels and a significant decrease in HDL-C levels in the skeletal muscle of zebrafish with T2DM (Figure 1g–j). To verify the stability of this model, we cultured the T2DM zebrafish that had been treated with high-glucose for 8 weeks in a normal breeding system for another 8 weeks and detected the insulin content in the skeletal muscle using IPGTT. Importantly, the T2DM-associated changes were still retained after the 8-week exposure to the normal breeding system (Figure 1k–m). Collectively, these results indicate that a high-glucose intervention for 8 weeks disrupts blood glucose homeostasis in zebrafish and affects lipid metabolism in the skeletal muscle.

### 2.2. Loss of Skeletal Muscle Mass and Decreased Proliferative Ability in T2DM Zebrafish

**Figure 2:**
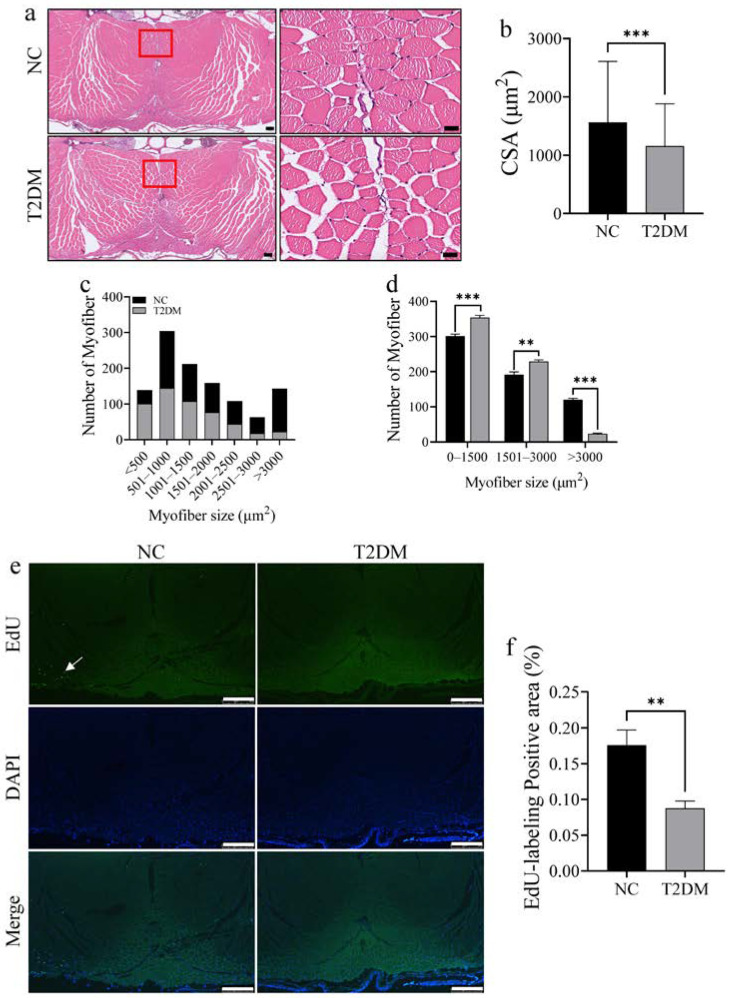
Skeletal muscle mass loss in zebrafish with type 2 diabetes mellitus (T2DM). (**a**) Hematoxylin and eosin (HE) staining of longitudinal sections of zebrafish tail skeletal muscle in each group, scale bar: 100 μm. The right side is an enlarged view of the red box on the left, scale bar: 20 μm. (**b**) Analysis of the total muscle fiber areas in each HE-stained section. (**c**) Analysis of the number of different muscle fiber areas in each HE-stained section. (**d**) Analysis of the difference in the muscle fiber area of the tail skeletal muscle in each group between 0 and 1500 μm^2^ (small), 1501 and 3000 μm^2^ (medium), and 3000 μm^2^ and above (large). (**e**) EdU staining intensity of the skeletal muscle of zebrafish, indicating muscle proliferation and regeneration. EdU-labeled areas are indicated by the white arrows, scale bar: 500 μm. (**f**) Statistical chart of the positive area of EdU labeling. ** *p* < 0.01, *** *p* < 0.001. (*n* = 3 samples/group).

To determine the effect of T2DM on skeletal muscle mass, we performed hematoxylin and eosin (HE) staining of the tail muscles and measured muscle fiber sizes in zebrafish in the two groups. T2DM induced skeletal muscle atrophy in zebrafish, as evidenced by a significant decrease in the overall cross-sectional area of muscle fibers, a significant increase in the cross-sectional area of small (<1500 μm^2^) muscle fibers, and a significant decrease in the cross-sectional area of large (>3000 μm^2^) muscle fibers (Figure 2a–d). To examine muscle cell proliferation, the fish were injected with an appropriate amount of 5-Ethynyl-2′-deoxyuridine (EdU, a thymidine analogue that is commonly used to label proliferating cells) via the abdominal cavity. Notably, there was a significant decrease in the EdU stained area in the skeletal muscle of zebrafish with T2DM (Figure 2e,f). Collectively, these results indicate that T2DM suppresses skeletal muscle regeneration in zebrafish.

### 2.3. T2DM Zebrafish Exhibit Increased Lipid Accumulation and Fibrosis in Skeletal Muscle

**Figure 3:**
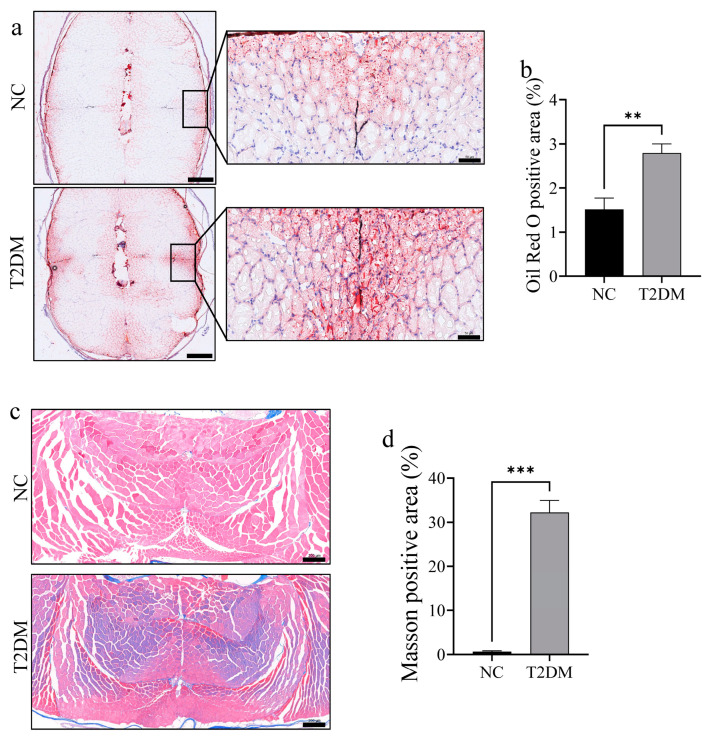
Lipid accumulation and fibrosis were significantly enhanced in the skeletal muscle of zebrafish with type 2 diabetes mellitus (T2DM). (**a**) Oil red O staining of skeletal muscle after 8 weeks of high-glucose treatment, with red indicating fat-positive staining. The image on the left is a low-magnification image (scale bar: 500 μm); the picture on the right is an enlarged view of the slow-twitch muscle area (scale bar: 50 μm). (**b**) Positive area of Oil red O staining; (**c**) Masson staining intensity in the skeletal muscle (scale bar: 200 μm); and (**d**) statistical chart of Masson staining positive area. ** *p* < 0.01, *** *p* < 0.001. (*n* = 3 samples/group).

Oil red O and Masson staining were performed to further observe changes in skeletal muscle mass in zebrafish with T2DM. Notably, there was a significant increase in lipid accumulation (mainly in the slow muscle part) (Figure 3a,b) and fibrosis (Figure 3c,d) in the skeletal muscle of zebrafish with T2DM. Overall, these results indicate that high-glucose treatment can significantly reduce skeletal muscle mass in zebrafish.

### 2.4. Skeletal Muscle Function Is Suppressed in T2DM Zebrafish

**Figure 4:**
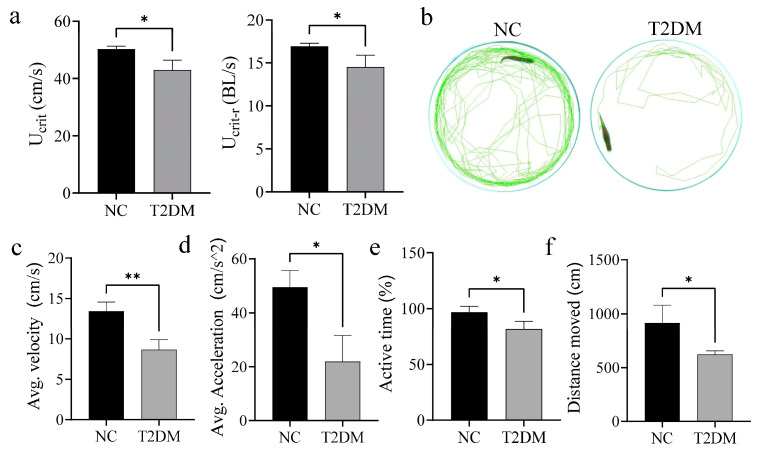
Type 2 diabetes mellitus (T2DM) impairs the motor ability of zebrafish. (**a**) T2DM significantly reduced the critical swimming speed (U_crit_) and relative critical swimming speed (U_crit-r_, U_crit_/body length (BL)) of zebrafish. (**b**) Image of zebrafish open-field experiment; (**c**) average speed of zebrafish open-field experiment; (**d**) average acceleration of zebrafish in the open-field experiment; (**e**) proportion of active time in the open-field experiment; and (**f**) total movement distance of zebrafish in the open field experiment. * *p* < 0.05, ** *p* < 0.01. (*n* = 6 samples/group).

The function of skeletal muscle is mainly to maintain body posture and movement through contraction and relaxation. To investigate the effect of T2DM on skeletal muscle function, we examined the movement ability of zebrafish. The critical swimming speed (U_crit_) in each group was measured using a Loligo System. Importantly, U_crit_ and relative critical swimming speed (U_crit-r_) were significant lower in the T2DM group than in the NC group (Figure 4a). Additionally, open-field experiments (Figure 4b) showed that the average swimming speed, average acceleration, active time, and total activity distance were significantly lower in the T2DM group than in the control group (Figure 4b–f). Collectively, these results indicate that chronic hyperglycemia affects the skeletal muscle mass of zebrafish and impairs exercise capacity.

### 2.5. T2DM Impairs Mitochondrial Function and Induced an Inflammatory Response in the Skeletal Muscle of Zebrafish

**Figure 5:**
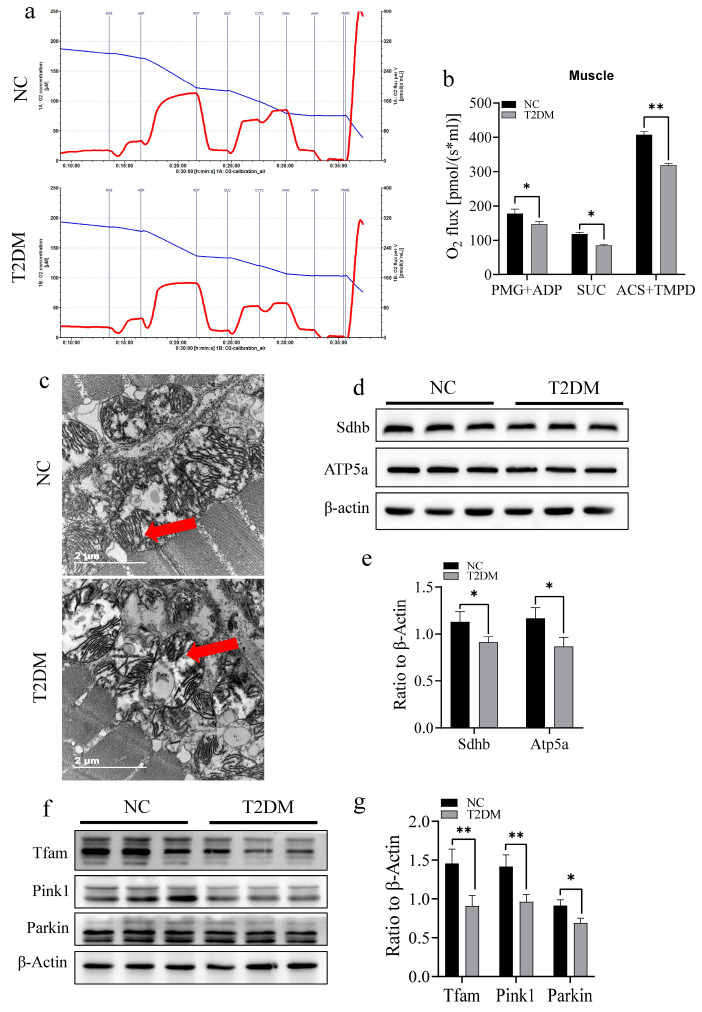
Type 2 diabetes mellitus (T2DM) causes mitochondrial dysfunction in the skeletal muscle of zebrafish. (**a**) Mitochondrial respiration test chart. The blue curve represents the oxygen content, while the red curve represents the curve of oxygen consumption change. (**b**) T2DM significantly reduces the respiratory capacity of skeletal muscle mitochondria, where PMG+ADP is the activity of complex I, SUC is the activity of complex II, and ACS+TMPD is the activity of complex IV. (**c**) Transmission electron microscope image of zebrafish mitochondria; T2DM induces the loss of intramitochondrial cristae (red arrow), scale bar: 2 μm. (**d**,**e**) T2DM downregulates the expression of key proteins of the mitochondrial respiratory chain complex in the skeletal muscle of zebrafish (Sdhb and Atp5a). (**f**,**g**) T2DM downregulates the expression of key proteins in muscle mitochondrial quality control (Tfam, Pink1, and Parkin). * *p* < 0.05, ** *p* < 0.01. (*n* = 3 samples/group).

**Figure 6:**
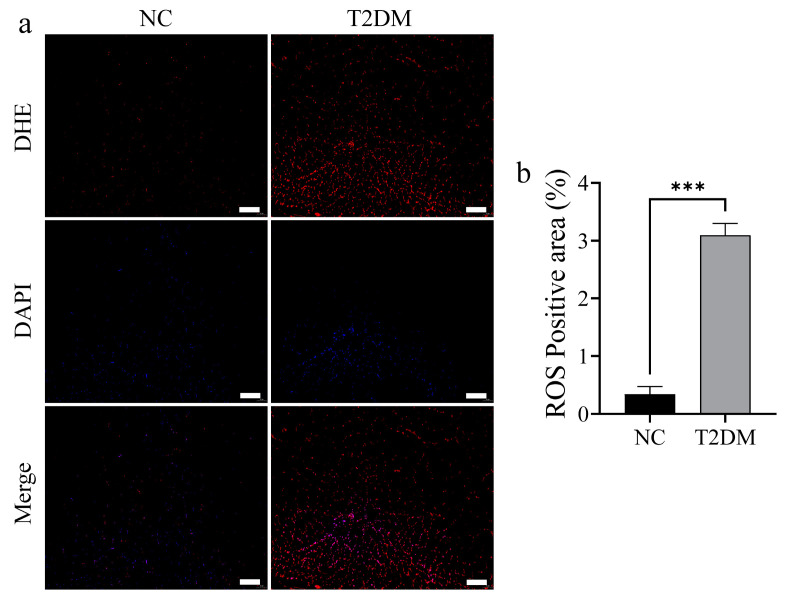
Type 2 diabetes mellitus (T2DM) induces inflammatory response in skeletal muscle. (**a**) Dihydroethidium (DHE) staining of zebrafish skeletal muscle, scale bar: 100 μm. (**b**) T2DM increases the accumulation of reactive oxygen species in the skeletal muscle. *** *p* < 0.001. (*n* = 3 samples/group).

Mitochondria provide energy for exercise through aerobic respiration. Therefore, we examined mitochondrial respiratory function to confirm whether the decrease in the exercise ability of zebrafish with T2DM was related to impaired mitochondrial function. High-glucose treatment significantly inhibited mitochondrial respiratory function in the skeletal muscle of zebrafish (Figure 5a). Specifically, the activities of complexes I (PMG + ADP), II (SUC), and IV (ASC + TMPD) in the respiratory chain were significantly inhibited (Figure 5b). Additionally, electron microscopy showed that high-glucose-induced T2DM caused a decrease in intramitochondrial cristae in skeletal muscle mitochondria in zebrafish (Figure 5c). Further molecular experiments indicated that high-sugar treatment significantly downregulated the expression of the ATP synthase protein Atp5a and Sdhb, the main functional protein of the mitochondrial respiratory chain complex II (Figure 5d,e), both of which are crucial for mitochondrial biogenesis. Additionally, high-sugar treatment significantly downregulated the expression of Tfam and the key mitophagy proteins Pink1 and Parkin (Figure 5f,g). Moreover, dihydroethidium (DHE) staining of the tail skeletal muscle showed increased ROS levels in the skeletal muscles of zebrafish with T2DM (Figure 6a,b). Overall, these findings indicate that T2DM adversely affects mitochondrial morphology and function.

### 2.6. T2DM Induces Mitochondrial Dysfunction via the miR-139a-5p/NAMPT Pathway

**Figure 7:**
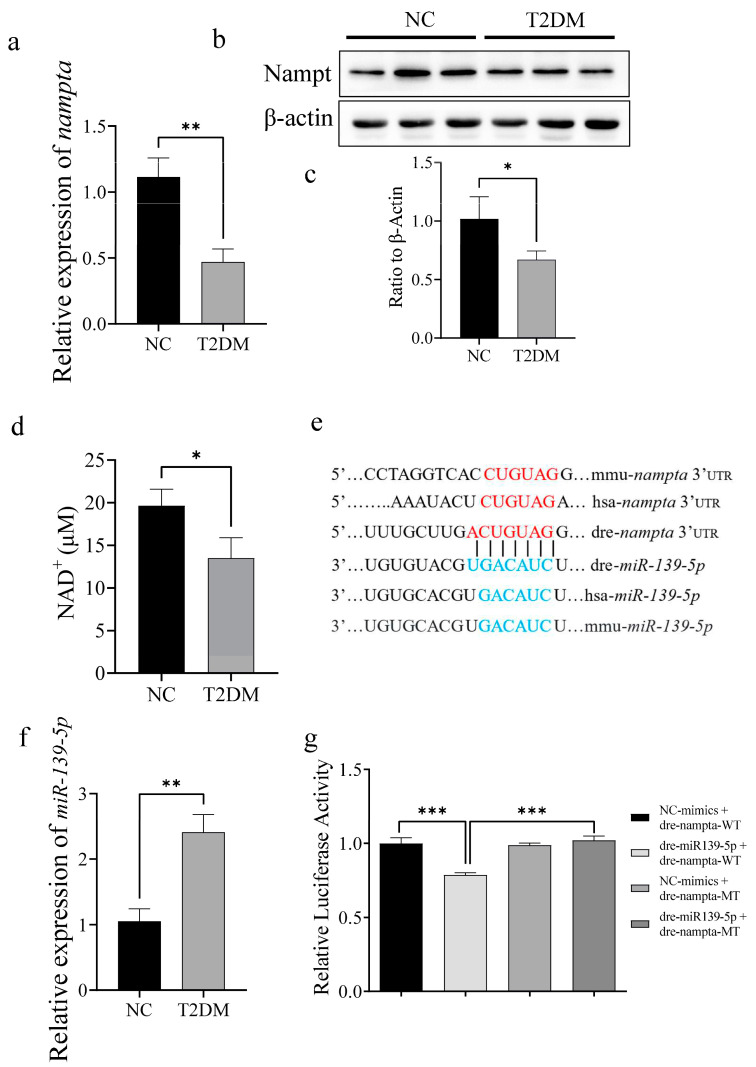
Type 2 diabetes mellitus (T2DM) inhibits NAMPT expression by upregulating miR-139a-5p. (**a**–**c**) T2DM significantly downregulates NAMPT mRNA and protein expression in the skeletal muscle of zebrafish. (**d**) T2DM significantly decreased NAD^+^ content in the skeletal muscle of zebrafish. (**e**) Using bioinformatics, we predicted that NAMPT is the target gene of miR-139a-5p; the red part is the seed region of NAMPT, and the blue part is the binding site between miR-139a-5p and the NAMPT seed region. (**f**) T2DM significantly upregulates miR-139a-5p in skeletal muscle. (**g**) The dual-luciferase reporter assay showed a regulatory relationship between miR-139a-5p and NAMPT. * *p* < 0.05, ** *p* < 0.01, *** *p* < 0.001. (*n* = 3 samples per group).

High-glucose treatment significantly downregulated the mRNA and protein expression levels of NAMPT in the skeletal muscle of zebrafish (Figure 7a–c). NAMPT is an essential rate-limiting enzyme involved in NAD^+^ production during mitochondrial respiration. High-glucose treatment significantly reduced the NAD^+^ content of the skeletal muscle of zebrafish (Figure 7d), indicating that T2DM affected NAMPT enzyme activity. Bioinformatics analysis suggested that miR-139-5p may target and regulate NAMPT (Figure 7e). Quantitative polymerase chain reaction (qPCR) showed that high-glucose treatment significantly upregulated miR-139-5p expression in the skeletal muscle of zebrafish (Figure 7f). Additionally, a dual-luciferase reporter assay indicated that miR-139-5p targeted and regulated NAMPT (Figure 7g). Collectively, these results indicate that high glucose may inhibit the expression and enzyme activity of NAMPT by upregulating miR-139-5p, thereby inducing mitochondrial dysfunction and decreasing exercise capacity in zebrafish.

## 3. Discussion

Insulin resistance is the core pathophysiology of T2DM [30], and it is the earliest metabolic abnormality observed in patients with prediabetes [31]. The skeletal muscle is the main site for insulin-mediated glucose disposal [32]. Diabetes can cause biological dysfunction in the skeletal muscle, including lipid infiltration, insulin resistance, inflammation, and mitochondrial dysfunction. Diabetic myopathy manifests as a loss of muscle mass and physical dysfunction [33,34]. In this study, we explored the pathogenesis of diabetic myopathy and provided a theoretical basis for the prevention and treatment of diabetes as well as for the design of exercise programs for exercise intervention in diabetic myopathy.

Aging is characterized by a loss of skeletal muscle mass and a decline in skeletal muscle function. However, patients with diabetes exhibit diabetes-related skeletal muscle decline even at a younger age [35]. In this study, zebrafish exposed to a high-glucose treatment for 8 weeks exhibited symptoms related to T2DM, including impaired glucose tolerance and a significant decrease in skeletal muscle insulin levels (since the blood volume of zebrafish is too small to detect serum insulin levels). Moreover, this model retained the high-glucose-induced characteristics of T2DM after 8 weeks of exposure to normal conditions. Additionally, we examined changes in the skeletal muscle of zebrafish with T2DM. Importantly, typical symptoms of T2DM were observed in the skeletal muscle of zebrafish fed a high-glucose diet, including decreased skeletal muscle mass, lipid accumulation and fibrosis in the skeletal muscle, reduced cross-sectional area of muscle fibers, decreased proliferation of muscle cells, and decreased exercise ability. Overall, these changes indicate the successful establishment of a zebrafish model of diabetic myopathy. Although skeletal muscle plays an important role in systemic metabolic regulation, research on its role in diabetic myopathy is limited. Hyperglycemia is the main cause of diabetic myopathy and mitochondrial dysfunction [36].

Research findings indicate that diabetes can significantly affect mitochondrial function in the skeletal muscle of individuals with diabetic myopathy [37]. The mechanisms underlying mitochondrial dysfunction are complex, and further research is necessary to elucidate the relationships among diabetes, mitochondria, and lipid metabolism. Mitochondria, which are the energy powerhouses of cells, primarily generate ATP through oxidative phosphorylation. However, impaired mitochondrial function affects energy production and leads to various metabolic disorders, especially in patients with diabetes. Our study revealed that high-glucose exposure significantly downregulated the activities of mitochondrial respiratory chain complexes I, II, and IV in the skeletal muscle of zebrafish, indicating a decrease in the ATP-generating efficiency of the mitochondria. Additionally, the morphology and internal cristae of the mitochondria were damaged. Although there are numerous mechanisms by which hyperglycemia leads to mitochondrial dysfunction, our study indicates that excessive ROS production may be involved in mitochondrial dysfunction. Excessive ROS production may damage respiratory chain electron transport by impairing the inner mitochondrial membrane, thereby affecting ATP production [38,39]. The reduced oxidative capacity of mitochondria affects the energy production. The weakened oxidative capacity is closely related to the pathogenesis of diabetes because mitochondrial dysfunction in pancreatic β cells can lead to reduced insulin secretion, thereby influencing glucose and lipid metabolism [40,41]. Mitochondrial dysfunction in pancreatic β cells induces hyperglycemia and abnormalities in lipid metabolism, typically manifested by elevated TG and LDL levels and reduced HDL levels [42,43]. Importantly, this abnormality in lipid metabolism is exacerbated following a decrease in insulin levels [44].

In diabetes, miR-139 is considered to play a crucial role in lipid metabolism and insulin resistance. Several studies have indicated that miR-139 expression is significantly elevated in patients with diabetes and in animal models. For instance, increased expression of miR-139 may be related to metabolic disorders and insulin resistance in murine models of diabetes [45]. miR-139 influences fat synthesis and breakdown by regulating the expression of genes related to lipid metabolism. Additionally, miR-139 can negatively regulate some key enzymes involved in lipid synthesis and breakdown, which may lead to the dysregulation of lipid metabolism [46]. Moreover, the high expression of miR-139 is also associated with the upregulation of proinflammatory factors, which affect the function of pancreatic β cells and their ability to secrete insulin, further exacerbating diabetes [47]. Considering the crucial role of miR-139 in diabetes, its potential as a biomarker has attracted research attention. Detecting plasma levels of miR-139 can provide new ideas for the early diagnosis and monitoring of diabetes. In the present study, miR-139-5p successfully bound to NAMPT, thereby affecting its expression in the skeletal muscle of zebrafish with T2DM. NAMPT activity is closely associated with mitochondrial health and function. Loss of NAMPT may lead to mitochondrial dysfunction, affecting energy metabolism. For example, NAMPT regulates insulin secretion in pancreatic beta cells in a process dependent on normal mitochondrial function and energy supply [20,48]. Additionally, NAMPT levels were significantly downregulated in mice fed a high-fat diet, which may lead to functional changes in mitochondria, thereby aggravating diabetes [20]. This is primarily because NAMPT, the rate-limiting enzyme in the NAD^+^ biosynthetic pathway, is responsible for converting nicotinamide into nicotinamide mononucleotide, an important intermediate in NAD^+^ synthesis [49]. This process is crucial for energy metabolism and affects the stress response and lifespan of cells. Additionally, NAMPT plays an important role in maintaining mitochondrial function. Mitochondria are the centers of cellular energy metabolism, and NAD^+^ plays an important role as a coenzyme in this process. NAMPT supports normal mitochondrial function by providing NAD^+^, thereby affecting cell survival and metabolism [50]. In the present study, we showed that miR-139-5p can regulate NAMPT in the skeletal muscle of zebrafish with T2DM. However, further studies are necessary to verify the regulatory roles of human miR-139-5p and NAMPT in T2DM-induced skeletal muscle disease.

As an alternative to rodent models, the zebrafish model has attracted considerable interest in diabetes research. Notably, studies have reported structural and physiological similarities in energy metabolism between zebrafish and humans [29]. Additionally, the zebrafish is a suitable model for the discovery and characterization of new diagnostic and therapeutic targets for metabolic diseases such as obesity [51], non-alcoholic fatty liver disease [52], atherosclerosis, and diabetes [53]. Notably, the zebrafish model of T2DM can be established using the following methods: the glucose solution immersion method [54,55], the high-fat diet induction method [56], the chemical induction method [57,58], and the combination of the high-fat diet and glucose immersion methods [59]. After overfeeding for one week, the fasting blood glucose level of zebrafish was approximately 1.5 times higher than that under normal feeding [60]. However, overfeeding for 6 weeks is necessary to induce these changes in mice [61]. Overall, these results indicate that zebrafish are more prone to glucose intolerance than mice, which may be due to the lower content of carbohydrates in the natural diet of zebrafish [62]. Therefore, excessive carbohydrates has a larger effect on glucose tolerance in zebrafish than in mammals, suggesting that zebrafish may be highly susceptible to T2DM [60,63].

In this study, we found that high-glucose treatment regulated the protein levels of NAMPT by upregulating the expression of miR-139-5p, resulting in mitochondrial dysfunction and decreased skeletal muscle mass in zebrafish. This study provides a theoretical foundation for subsequent research on the molecular mechanisms of exercise intervention in diabetic myopathy. However, it is important to acknowledge that this study has some limitations. For example, although we provided preliminary evidence regarding the regulatory relationship between miR-126-5p and NAMPT, further studies on the role of miR-139-5p/NAMPT in diabetic myopathy are necessary to validate our findings. Additionally, although mitochondrial function in skeletal muscles is similar between zebrafish and rodent models, further validation is essential to contextualize our findings within the broader scope of metabolic research. Moreover, establishing a clear link between findings in zebrafish and rodents would significantly enhance the applicability of our research. Addressing these limitations not only strengthens the current report but also highlights potential avenues for future investigations.

## 4. Materials and Methods

### 4.1. Experimental Subjects and Grouping

Healthy male zebrafish (*Danio rerio*; wild-type AB strain; (*n* = 100) aged 6 months) sourced from the China Zebrafish Resource Center and housed in an optimal environment (28 ± 0.5 °C, relative humidity ≤ 60%, and 14:10 h light/dark cycle). The fish were subjected to a 1-week adaption period and fed fresh brine shrimp daily on a regular schedule. After the adaptive feeding period, the zebrafish were randomly divided into two groups: Normal Control (NC, *n* = 50) and T2DM model (T2DM, *n* = 50). All zebrafish were reared in a breeding system. Fish in the NC group were fed fresh brine shrimp thrice a day, whereas the T2DM group was fed high-sugar brine shrimp soaked in 50% glucose. In the evening, zebrafish in the T2DM group were soaked in 5 L of breeding water containing glucose (1% in the first week, 2% in the second week, 3% in the third week, 4% in the fourth week, and 4% from the fifth to the eighth weeks) for 14 h, whereas those in the NC group were raised in normal breeding water. This study was approved by the Laboratory Animal Use Ethics Committee of Hunan Normal University (Approval No.: 2018-046).

### 4.2. Zebrafish Exercise Capacity Test

The U_crit_ represents the maximum continuous swimming speed. Before formulating an exercise plan, it is necessary to measure the U_crit_ and active metabolic rate to calculate the optimal swimming speed (U_opt_) required during aerobic exercise. Before the experiment, the fish were anesthetized with 40 mg/L of MS-222 (using culture water), and their standard body length (BL) (cm) and body weight (BW) (g) were measured. Thereafter, the exercise capacity test was performed as previously described [64].

### 4.3. Intraperitoneal Glucose Tolerance Test and Whole-Body Biochemical Analysis

After fasting for 24 h, the fasting blood glucose level of the experimental fish was measured using a micro blood glucose meter (yuwell, Danyang, China). Thereafter, the fish were injected with a glucose solution via the abdominal cavity at a dosage of 0.5 mg of glucose per gram of body weight, and blood glucose levels were measured at 30, 90, and 180 min post-injection.

For the biochemical analysis, the zebrafish were sacrificed and the tail skeletal muscle was dissected, homogenized with normal saline, and centrifuged to collect the supernatant. Thereafter, the triglyceride (TG), total cholesterol (TC), high-density lipoprotein-cholesterol (HDL-C), and low-density lipoprotein (LDL-C) contents of the skeletal muscle were determined using specific kits (Njjcbio, Nanjing, China). Additionally, the insulin level in the skeletal muscle was measured using an ELISA kit (MEIAO BIOTECH, Shanghai, China).

### 4.4. Sample Collection

After the experiment, fish were anesthetized with MS-222 and cut into dorsal and caudal sections along the longitudinal axis from the apex of the dorsal fin to the cloaca to collect relevant tissues. Tissues for morphological analysis were fixed in 4% paraformaldehyde and examined using an electron microscope. Additionally, excess skin tissue was carefully removed from the remaining portion of the skeletal muscle using a surgical scalpel and washed in 1× phosphate-buffered saline (PBS). After removing the bones, the samples were rapidly frozen in liquid nitrogen and stored in an ultra-low temperature freezer at −80 °C.

### 4.5. Mitochondrial Respiration Detection

Mitochondrial respiration was detected using an Oroboros Instruments O2K instrument (Oroboros Instruments GmbH, Innsbruck, Austria). The procedure for mitochondrial respiration detection was performed according to a previously published paper [65]. Briefly, 4 mg of caudal skeletal muscle samples were ground on ice using the MIR05-kit and placed in a test chamber. After achieving the baseline level, 5 μL of 2 M pyruvate, 10 μL of 400 mM malate, 10 μL of 2 M glutamate, 15 μL of 500 mM ADP, 1 μL of 1 mM rotenone, 20 μL of 1 M succinate, 5 μL of 4 mM cyt C, 5 μL of 2 M malonic acid, 1 μL of 5 mM antimycin A, 5 μL of 0.8 M ascorbate, and 5 μL of 0.2 M *N,N,N′,N′*-Tetramethyl-*p*-phenylenediamine dihydrochloride (TMPD) were added to the sample. Data were collected and analyzed.

### 4.6. Western Blotting

Western blot analysis was performed to examine the expression of specific proteins following a previously described protocol [64]. The primary antibodies used were ACTIN, SDHB, ATP5a, TFAM, PINK1, PARKIN, NFκb, and NAMPT (1:2000, Servicebio, Wuhan, China).

### 4.7. Detection of Tissue NAD^+^ Content

Briefly, fresh skeletal muscle tissue (30 mg) was extracted with an extraction solution at a volume nine times the weight of the tissue. Specifically, the tissue was cut into small pieces, homogenized in an ice-water bath, and centrifuged at 12,000× *g* for 10 min to obtain the supernatant. Finally, the NAD^+^ content was determined using the NAD^+^/NADH Assay Kit with WST-8 (S0175, Beyotime, Shanghai, China).

### 4.8. Transmission Electron Microscopy

Briefly, fresh skeletal muscle tissue was fixed in 2.5% glutaraldehyde solution for 6–12 h, washed with PBS, and fixed in 1% osmium tetroxide for 1–2 h. Thereafter, the samples were cut into ultrathin sections, stained with lead and uranium (electron staining), and observed under a JEOL JEM1400 transmission electron microscope (JEOL, Tokyo, Japan). Images were captured using a Morada G3 digital camera (EMSIS GMBH, Münster, Germany).

### 4.9. Dual-Luciferase Reporter Gene Assay

To verify the relationship between miR-139-5p and NAMPT, we constructed the binding site of miR-139-5p and dre-nampta as well as the binding site mutants into dre-nampta-WT (GCTAGCTAAATCATTTAACCCAGCGTCAAAATGCACACGTCTTTGCAGGAATGGAGACGTTTATGTGTGTGTAAAAACGGCCAGGAGTTTTGGCTTTGCTTGACTGTAGGACTGAAAGCTAATCGTCCAGTGTTTGTCGAATGTGAATGTCGTGTGTAGAGTTTTTCACTTTATCAGAGGATTAATCTATTCCTTTATGCAAAGAGCTCGAG) and dre-nampta-MT (GCTAGCTAAATCATTTAACCCAGCGTCAAAATGCACACGTCTTTGCAGGAATGGAGACGTTTATGTGTGTGTAAAAACGGCCAGGAGTTTTGGCTTTGCTTGATCACGAGACTGAAAGCTAATCGTCCAGTGTTTGTCGAATGTGAATGTCGTGTGTAGAGTTTTTCACTTTATCAGAGGATTAATCTATTCCTTTATGCAAAGAGCTCGAG) plasmids, respectively. Thereafter, the native control miRNA (NC-mimics: Sense 5′-UCACAACCUCCUAGAAAGAGUAGA-3′, Anti-sense 5′-UACUCUUUCUAGGAGGUUGUGAUU-3′), wildtype seed sequence of NAMPT (dre-nampta-WT), miR139-5p mimics (dre-miR139-5p mimics: Sense 5′-UCUACAGUGCAUGUGUCU-3′, Anti-sense 5′-ACACAUGCACUGUAGAUU-3′), and NAMPT seed sequence with mutations (dre-nampta-MT) were co-transfected into 293T cells using the following combinations: NC-mimics + dre-nampta-WT, dre-miR139-5p-mimics + dre-nampta-WT, NC-mimics + dre-nampta-MT, and dre-miR139-5p-mimics + dre-nampta-MT. Finally, the relative light unit (RLU) value obtained by measuring the firefly luciferase was divided by the RLU value obtained by measuring the Renilla luciferase (internal reference). The activation levels of the target reporter genes in different samples were compared according to the obtained ratios.

### 4.10. EdU Cell Proliferation Assay

Briefly, 20 μL of 50 mg EdU (prepared with normal saline) was injected into the abdominal cavity of zebrafish. Approximately 12 h later, the fish was sacrificed and dissected to collect the complete tail skeletal muscle. Thereafter, the sample was fixed with 4% paraformaldehyde, embedded in paraffin, cut into sections, and stained with EdU using BeyoClick™ EdU Cell Proliferation Kit with Alexa Fluor 488 (C0071S).

### 4.11. Statistical Analysis

All data are presented as mean ± standard error of the mean (SEM). Significant differences between groups were determined using the unpaired sample *t*-test. Graphs were plotted using GraphPad Prism 8 (Boston, MA 02110, USA). Statistical significance was set at *p* < 0.05. All experiments were repeated thrice.

## Data Availability

Data will be available upon request from the corresponding author.
